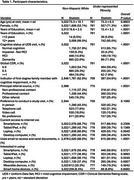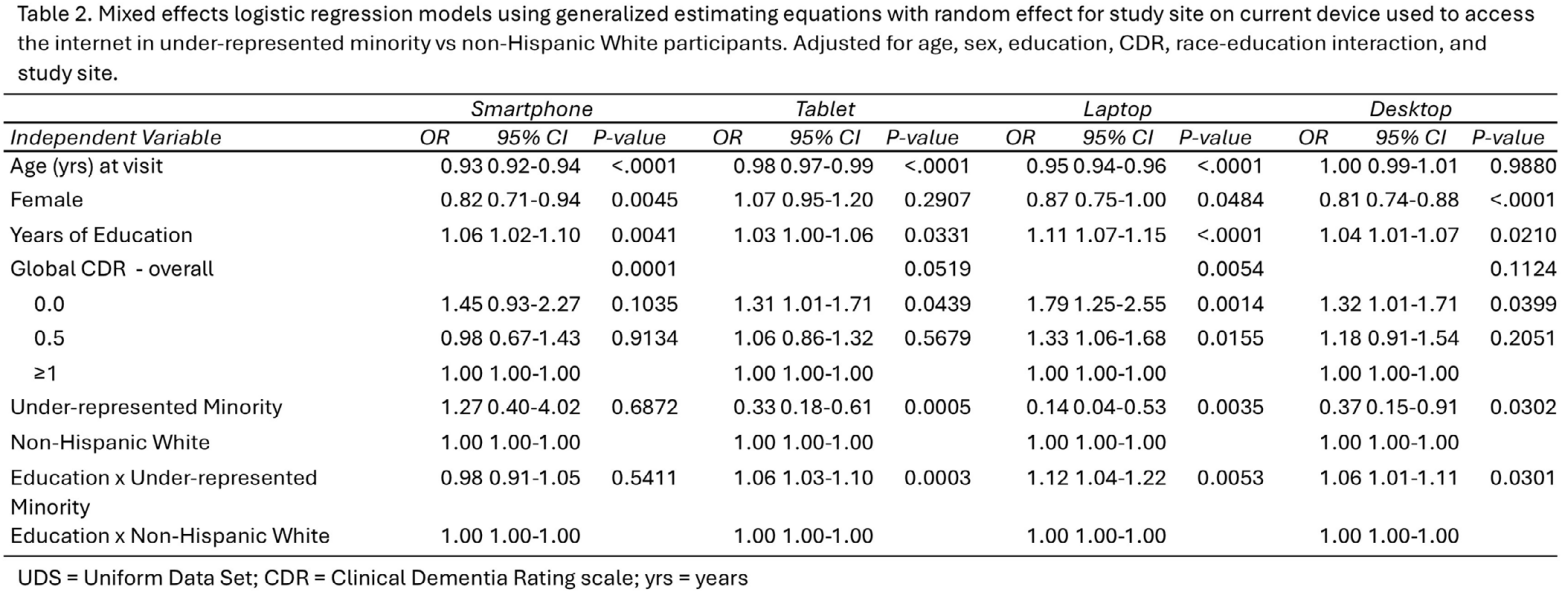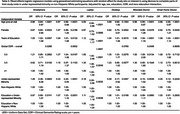# Patterns of technology access and preferences for remote assessments across groups at Alzheimer's Disease Research Centers

**DOI:** 10.1002/alz70858_099484

**Published:** 2025-12-25

**Authors:** Carol K Chan, Kathleen A. Lane, Sujuan Gao, Omolola A Adeoye‐Olatunde, Patrick Shih, David K Johnson, Andrew J. Saykin, Sophia Wang

**Affiliations:** ^1^ Case Western Reserve University School of Medicine, Cleveland, OH, USA; ^2^ Indiana University School of Medicine, Indianapolis, IN, USA; ^3^ School of Medicine, Indiana University‐Purdue University at Indianapolis, Indianapolis, IN, USA; ^4^ Purdue University College of Pharmacy, West Lafayette, IN, USA; ^5^ Luddy School of Informatics, Computing, and Engineering at Indiana University Bloomington, Bloomington, IN, USA; ^6^ University of California, Davis, Davis, CA, USA; ^7^ Indiana Alzheimer's Disease Research Center, Indiana University School of Medicine, Indianapolis, IN, USA

## Abstract

**Background:**

Using remote assessments for Alzheimer's Disease and Related Disorders (ADRD) studies can have advantages, such as providing research opportunities to individuals who might otherwise be excluded due to geographical distances, transportation difficulties, and physical frailty. As studies adopt remote assessment modalities, however, people at highest risk of ADRD may be less likely to use the internet, own electronic devices, and be comfortable with technology utilization.

**Method:**

These analyses included data obtained through the National Alzheimer's Coordinating Center Uniform Data Set from 3,803 participants across 17 Alzheimer's Disease Research Centers in the United States who completed the Technology Access Survey between July 2^nd^, 2020 and April 26^th^, 2023. Participants were categorized as either White or Other Race or Ethnicity. Mixed effects logistic regression models using generalized estimating equations with random effect for study site were used to examine the association of education, race and ethnicity, and education x Other Race or Ethnicity interaction with (1) device use and (2) device preferences for remote assessments. The analyses were adjusted for age, sex, cognitive status, and study site. Significance was set at *p* <0.05.

**Result:**

Descriptive statistics are shown in Table 1. Participants with more years of education had greater access to the internet across all devices (Table 2). Other Race or Ethnicity participants had lower odds of access to tablet, laptop and desktop computer compared to White participants. There was a significant interaction between Other Race or Ethnicity and education for use of tablet, laptop and desktop computer, where the effect of higher education was greater in Other Race or Ethnicity than White participants. A similar pattern of results was observed for interest in using a smartphone, tablet, laptop or desktop computer to complete parts of their study visit from home (Table 3).

**Conclusion:**

These findings suggest that education has a role in racioethnic differences in technological access and preferences. Future ADRD studies utilizing remote assessments should consider these patterns to inform study design and potential selection of populations studied.